# Does the disclosure of medical insurance information affect patients’ willingness to adopt the diagnosis related groups system

**DOI:** 10.3389/fpubh.2023.1136178

**Published:** 2023-08-21

**Authors:** Zhenni Shi, Qilin Zhang, Xiaofeng Wang

**Affiliations:** ^1^School of Political Science and Public Management, Wuhan University, Wuhan, China; ^2^College of Management, Shenzhen University, Shenzhen, China

**Keywords:** medical insurance information, DRG, patients’ willingness, risk perception, trust perception

## Abstract

**Introduction:**

Medical insurance information disclosure is not only a direct way for the public to understand and master social insurance information and resource use benefits, but also an important way for the public to participate in medical service governance and supervision. Some studies have shown that information disclosure can significantly reduce the risk perception of user groups, strengthen their trust and reduce the negative impact of information asymmetry.

**Methods:**

Based on risk perception and trust perception theories, this paper focuses on the mechanisms influencing patients’ attitudes in the process of implementing a Diagnosis Related Groups payment system. Using medical insurance information disclosure from a governance perspective as the research object, the impact of medical insurance information disclosure on patients’ willingness to adopt the Diagnosis Related Groups payment system was analyzed by means of a questionnaire survey, Data analysis and hypothesis testing *via* SPSS while the mechanism of the impact of medical insurance information disclosure on patients’ attitudes was explored in depth.

**Results:**

It was found that medical insurance information disclosure had a significant positive effect on patients’ trust perceptions and a significant negative effect on patients’ risk perceptions. The more comprehensive information patients received, the stronger their trust and the lower their perceived risk.

**Discussion:**

This paper conducts an empirical study from patients’ perspective, broadens the scope of research on medical insurance Diagnosis related groups, enriches the application of risk perception and trust perception theories in the medical field, and provides management suggestions for medical institutions in the management of medical insurance information disclosure.

## 1. Introduction

The world’s population is aging rapidly. The World Health Organization says the world’s population over 60 will double to 2.1 billion people by 2050. In China, the older adult population is expected to grow to 366 million by 2050, with the proportion of the older adult population rising to 26.1 percent (1). All countries are facing the major challenge of population aging, and organizations are actively exploring new programs to address this demographic shift. The aging of population has brought great economic burden to the society at the same time, it also brings great challenges in the aspects of medical insurance (2). Compared with younger groups, the older adult have reduced immune function and often suffer from various chronic diseases, which puts great economic pressure on families and society and seriously affects their quality of life (3). As the number of older adult population increases year by year, the demand for medical services and resources also increases (4). Rising health care expenditures place a heavy financial burden on individual patients and national health insurance plans. In China, this burden is largely caused by the previous payment system (5). In the past, payment by service item and other medical insurance payment methods generally believe that the higher the price, the better the quality of services, leading to the unreasonable increase of medical insurance fund expenditure, resulting in the waste of medical insurance fund (6). Therefore, it is urgent to strengthen the reform of medical insurance payment and provide quality medical services for medical insurance enrollees, especially older adult enrollees (7). In November 2021, the National Medical Insurance Administration issued the “Three-Year Action Plan for the Reform of Diagnosis related groups (DRG) Payment Method,” which clarified that “after the overall planning area starts the DRG (Diagnosis Related Groups, payment based on disease diagnosis-related groups) payment reform work, the DRG payment medical insurance fund expenditure will be realized according to the three-year arrangement (8). This is accounting for the goal of reaching 70% of the hospitalization medical insurance fund expenditure in the overall planning area (9).

Diagnosis related groups were derived from the reform of the health care expenditure system in the United States in the 1980s, and have gradually been used worldwide (10). DRG payment is currently recognized as one of the most effective and scientific medical insurance payment methods in the world (11). As of October 2022, DRG actual payment in 30 pilot cities has covered 807 medical institutions, and the coverage rate of tertiary hospitals has reached 57.4% (12). DRG payment is the core of medical insurance payment reform, an important measure to deepen the reform of the medical system and has far-reaching significance for balancing the quality of medical services and the use of medical insurance funds (13). The implementation of the DRG payment system means that the living environment of public hospitals is undergoing profound changes, which brings great uncertainty to the industry competition they face (14). It will comprehensively improve medical quality, implement refined management, and strengthen risk management (15). New requirements and challenges have been raised. The most important role played by the DRG medical insurance reform is to provide scientific basis for the analysis and evaluation of the cost, performance and quality of medical services, help patients understand the corresponding medical service process of the hospital, strengthen supervision, and realize the transparency of medical service management (16).

At present, although there are a large number of literatures to study the willingness of medical personnel and management departments to adopt and use the DRG medical payment system (17). However, there is no literature on the willingness of medical insurance users and patients to adopt the DRG medical payment system and explore its influencing factors on their willingness to adopt. Compared with the existing literature, this study will conduct an in-depth study on the attitude and willingness to adopt the DRG medical payment system from the perspective of patients. The patient’s willingness and attitude to use play an important role in the successful promotion and use of the DRG medical payment system.

After research, trust and risk perception are important factors that affect patients’ willingness to comply with and adopt the DRG payment system. Therefore, it is of great practical significance to explore the reasons for the low willingness of patients to adopt the DRG payment system and seek effective measures to increase the willingness of patients to adopt the DRG payment system. However, in countries that have adopted DRG for payment, there are also characteristics of poor health insurance information disclosure, DRG has changed the behavior and habits of patients, making patients do not understand the existing health insurance system, resulting in information asymmetry (18). Previous studies have shown that information disclosure can increase public trust and reduce public risk perception. Under the framework of modern medical governance, the disclosure and transparency of medical insurance information will help patients know the source and whereabouts of medical income and expenditure, and facilitate the supervision of hospital behavior by patients and medical insurance users, which is conducive to standardizing hospital medical behavior and enhancing patients’ confidence. Trust and reduce risk perception of patients. In a highly regulated medical environment, patients are more inclined to adopt the highly secure DRG payment system. As a relatively scientific and rational method of medical cost management and quality evaluation, DRG is a medical management tool, and the most important thing is to realize the win–win situation of medical insurance and patients in hospital management practice (19). However, patient groups are not concerned about this new health care model. The asymmetry of information may lead to the fact that patients will not easily choose the new health insurance payment model. For this reason, this study from the perspective of medical insurance information disclosure and medical insurance information transparency, it is an important measure to deepen the reform of the medical system to investigate the factors that affect the willingness of patients to adopt the DRG payment system, and to explore effective ways to improve the adoption of the DRG payment system by medical insurance users and patients in my country. It is of far-reaching significance to balance the quality of medical services and the use of medical insurance funds.

## 2. Literature review and research hypothesis

### 2.1. Diagnosis related groups

Diagnosis related groups (DRG) is a system that divides patients into multiple diagnostic groups for management based on their age, diagnosis, complications and complications, severity of disease, treatment and outcome, and resource consumption. It is an important means to establish a new operation compensation mechanism for public hospitals, improve the problem of unreasonable increase of medical costs, gradually promote hierarchical diagnosis and treatment, promote the transformation of service mode, and finally realize a win–win situation among medical insurance and patients. Previous studies on DRGS mostly focused on the analysis of DRG payment policy itself and its impact on medical institutions and medical services. Table 1 provides a summary of the existing literature on DRG payment systems.

**Table 1:** Diagnosis related groups literature review.

References	Research perspective	Research variable	Conclusion
(20)	DRG	Medical services are the main influencing factors	The DEMATEL method is used to identify the factors affecting the overall performance of medical service, and it is suggested to pay attention to the ability of medical service providers when selecting or using DRG
(21)	patient	Composition of medical expenditure	Based on the decision tree model, the process of hospitalization, surgery, diagnosis and treatment of colorectal cancer patients is managed, reducing the financial burden of patients
(22)	patient	Hospital caseload	Inpatient medical costs and average length of stay vary significantly in hospital concentration index, and the extension of DRG payment system to hospitals will negatively impact their total sales
(23)	DRG	Intensive care and nursing services	The shortage of qualified intensive care nurses and doctors is the biggest threat to intensive care and recommendations are made to address this problem
(24)	DRG	DRG costs	LM is a superior method to detect both low and high outliers for DRG costs, thereby improving the efficiency and effectiveness of DRG prospective payment systems and equity of healthcare
(25)	doctor	Medical practice	The implementation of DRGS has transformed medical practice into a process of cost-effectiveness optimization
Our research	patient	Disclosure of medical insurance information, willingness to adopt	Trust and risk perception are important factors affecting patients’ willingness to comply with the adoption of DRG payment system

It can be found that few scholars discuss the DRG system from the perspective of patients. Therefore, this study analyzes the DRG system from the perspective of patients, trying to broaden the scope of research in this field.

### 2.2. Disclosure of medical insurance information

Information disclosure refers to the system in which an organization actively discloses information to the public or to specific individuals or organizations through certain forms and procedures in the course of daily operations (26). Similar expressions include “information disclosure,” “information transparency,” etc. “Wait. The “Guiding Opinions on Actively Promoting the Reform of Medical Care, Medical Insurance, and Medical Linkage” pointed out that the reform of the medical and health system should be deepened, the medical care, medical insurance, and medical linkage should be implemented, and the role of medical insurance in medical reform should be fully utilized (27). In order to better play the role of medical insurance in the medical reform, it is necessary to let the public understand the medical insurance policy (28). The disclosure of medical insurance information by hospitals is an important way for the public to obtain information (29). The disclosure of hospital medical insurance information can let the public understand the medical insurance policy and help the public make reasonable decisions when seeking medical treatment (30). The disclosure of medical insurance service information can allow the public to enjoy medical insurance benefits conveniently and improve the public’s medical experience (31). At the same time, the disclosure of medical insurance information can increase the trust of patients and reduce the risk perception of patients to the hospital (32). Therefore, hospital administrators should pay attention to the disclosure of medical insurance information (33).

With the rapid development of Internet information technology, the disclosure of medical insurance information will be an important part of the reform of my country’s medical and health services, and an important starting point for the government to supervise hospitals (34). It is an important guarantee for patients to trust medical institutions (19). The health administrative department should formulate corresponding policy documents to clarify the specific requirements for hospital medical insurance information disclosure, so that hospitals follow a unified disclosure standard, and at the same time strengthen the supervision of information disclosure, and increase the motivation of hospitals to disclose information (35). In 2022, a survey of the “National People’s Trusted Model Hospital” announced by the Chinese Hospital Association found that 51.5% of the official websites of hospitals disclosed medical insurance policies, and 22.7% of the official websites of hospitals disclosed medical insurance details (36). Hospital medical insurance information disclosure is an important way for the public to obtain medical insurance information, and incomplete public information is not conducive to the public’s comprehensive understanding of medical insurance policies and services. In order to provide patients with better medical insurance services and medical experience, hospitals need to disclose complete medical insurance information and make medical insurance information transparent (37). The disclosure of medical insurance information will also affect patients’ trust in wishes and risk perception.

### 2.3. Trust perception theory

Trust Perception Theory is a theory that studies how people generate and perceive trust and mistrust, and it emphasizes the process of perceptual and cognitive interactions between individuals. The theory argues that the interaction between individuals and their environment can lead individuals to develop trust, and at the same time, specific environments can change their trust perceptions (38). Trust is a complex psychosocial phenomenon that involves multiple dimensions and dimensions. Wang Menghan considers trust as an attitude that someone’s behavior or the order around them is in accordance with his or her wishes, and believes that the technical competence of a certain person is one of the important factors affecting trust (30).

The doctor–patient relationship is the most fundamental social relationship in health care (39). For a long time, the frequent occurrence of doctor-patient disputes has led to the deterioration of the doctor-patient relationship, which has caused widespread concern in the society (35). The doctor-patient disputes have brought serious negative impacts to the health care system, causing both hospitals and patients to fall into the “access paradox trap.” The root cause of doctor-patient conflict is lack of trust (40). According to Powell, “trust can be used to solve complex practical problems more quickly and with less effort than using authority to limit or use predictions and other methods.” The degree of transparency of health care providers’ information about health care is an important factor that influences residents’ trust in hospitals (41) Chunnian and Lingyu (38) identified medical information disclosure as an important factor influencing doctor-patient trust. Jinshu et al. considered hospital disclosure of health care information as an important factor influencing patients’ willingness to receive follow-up services and cooperation from hospitals (42). In the doctor–patient relationship, a higher level of patient trust in the hospital can help doctors carry out better clinical treatment and increase patients’ sense of security, which is especially important for patients’ active cooperation and disease recovery (43). Patients must first trust the hospital in order to actively cooperate with it and cooperate with the relevant policies introduced by the hospital (32). Several studies have found that patients generally have a pre-determined distrust of hospitals (44). This mistrust exists before the actual effective communication and information interaction between the doctor and patient, which affects the interests of both the doctor and the patient.

### 2.4. Risk perception theory

Currently, there are two relatively well-established theories for the study of risk perception, namely risk culture theory and risk psychometric theory (45). The school of risk culture theory, led by Douglas, defines the degree of risk perception of individuals mainly through common cultural communication (37). Different individuals have different risk perceptions in the face of different environments and cultures. The school of risk psychometrics led by Slovic mainly uses psychological knowledge to measure individual risk perceptions and uses psychometric paradigms to measure individual risk perceptions (46). Risk perception theory studies people’s subjective psychological feelings from an individual perspective (47). Through the limited rational behavior people exhibit, it is reasonable to expect people’s perceived assessment and perception of risk in different environments and scenarios, and what risky decisions people will make (35). Since then, with the continuous development of risk theory, many scholars have extended the concept of risk perception to various degrees (48). Bauer understands risk perception as the inability of consumers to accurately identify the good or bad of their behavioral decisions, which leads to uncertainty about the outcome (49). Slovic (50) formulated risk perception as an individual’s judgment of risk given limited or uncertain knowledge of the information environment.

Risk perception theory emphasizes the importance of people’s subjective perceptions of risk and their perceptions of the environment. It has been applied in many fields, such as health, finance and environmental issues, to help people more accurately assess possible risks and make more informed decisions. In healthcare, there is a relationship between patients’ risk perception and patients’ behavioral decisions (51). In studying the influence of risk perception on patients’ behavioral decisions to enroll in insurance, Sun Jiaxin et al. (52) focused on patients’ risk perceptions of hospitals. It was found that the lower the patients’ risk perception of the hospital, the more patients participated in hospital decision making and actively cooperated with hospital medical behaviors.

### 2.5. Willingness to adopt

Yang Weizhong (49) found that patients’ risk perception level and patients’ trust in the hospital have an important impact on whether patients actively cooperate with the hospital. Chen Xinjian and Wei Yuanyuan (53) combined field survey data and used empirical analysis methods to study the risk management strategies perceived by hospital patients. The research results showed that there was a significant inverse relationship between patients’ risk perception level and the degree of hospital information disclosure. Xue Wentian’s (54) study found that patients’ risk perception is low, and the higher the trust level, the stronger the patient’s willingness to actively cooperate with the hospital and take hospital-related measures.

In this study, patients’ willingness to adopt the DRG payment system is mainly affected by two factors: patients’ trust and risk perception. The higher the patient’s trust in the hospital, the stronger the patient’s willingness to adopt the DRG payment system. The stronger the risk perception felt by the patient, the lower the patient’s willingness to adopt the DRG payment system.

### 2.6. Research hypothesis

The disclosure of medical insurance information can make the whole process of medical institutions from management, operation to service open and transparent, so as to improve the supervision of the public and improve the quality of their medical services (55). Some scholars have pointed out that the information disclosure content, channels and effects of an organization have a positive impact on the trust of the organization (50). If the organization discloses false information or the information disclosed is incomplete, it will cause public distrust of the organization. Another study pointed out that the imbalance between the supply and demand of medical service information is the key reason for the lack of mutual trust between doctors and patients in recent years (56). Strengthening the disclosure of medical service information to meet the information needs of patients can reduce the degree of information asymmetry between doctors and patients and reshape the mutual trust between doctors and patients (57). In addition, Xiaokang et al. (47) and others explored the cognitive mechanism of individuals’ perception of medical information risk based on the theory of empirical analysis and processing and word-for-word processing. Therefore, this study hypothesizes:

*H*1: Medical insurance information disclosure can improve patients' trust perception.

*H*2: Medical insurance information disclosure can reduce patients' risk perception.

In terms of risk perception, Chunnian and Lingyu (58) found through empirical research that user perceived risk has a direct impact on user experience and continuous use intention of emergency website information services, and Ruixian and Mengjun (59) found that user perceived risk will significantly affect social software users. Yanan and Chaohua (60) found that perceived financial risk and perceived time risk are the main variables that affect user satisfaction and continued use intention through the user satisfaction of online medical and health websites. Sun et al. (61) also explored the direct effect of risk perception on usage behavior. The studies of the above scholars all reflect that risk perception affects users’ willingness to use to a certain extent. Secondly, in terms of trust perception, Mengxuan and Weihua (28) built a model of factors influencing public willingness to use Internet medical service platforms based on trust theory, and verified through analysis that trust has a direct and positive impact on public willingness to use. Yanan & Chaohua (62) constructed a relationship model between the quality of government electronic information services and the public’s willingness to continue to use, pointing out that perceived trust can have a positive impact on the willingness to continue to use. The research of the above scholars reflects that trust perception affects users’ willingness to use to a certain extent. Based on this, in order to explore the influencing factors of patients’ willingness to adopt DRG, this study focuses on the internal perception state of patients, and puts forward the hypothesis:

*H*3: Improving patients' trust perception is conducive to improving patients' willingness to adopt DRG.

*H*4: Reducing the risk perception of patients is conducive to improving patients' willingness to adopt DRG.

**Figure 1:**
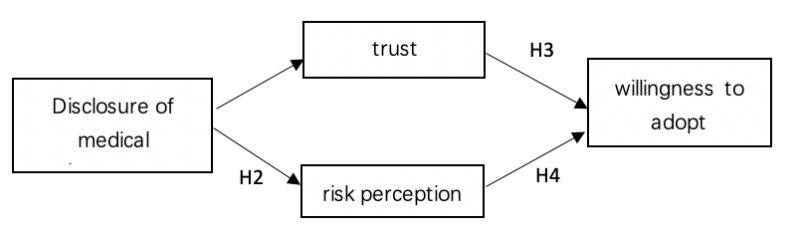
Framework diagram of theoretical model.

As shown in Figure 1, the theoretical model proposed in this study assumes that the disclosure of medical insurance information will increase patients’ trust perception, reduce patients’ risk perception, and then increase patients’ willingness to adopt DRG.

## 3. Data and method

### 3.1. Scale design

On the basis of referring to the existing research, combined with the development status of medical insurance DRG payment, this study improved the relevant scales and designed a questionnaire on the willingness of medical patients to adopt the medical insurance DRG system. The questionnaire is mainly composed of two parts, the first part is the basic information of the patient, and the second part is factor analysis, which is set as a scale question and selected as a five-point Likert scale to measure, 1 means strongly disagree and 5 means strongly agree. The measurement of medical insurance information disclosure mainly refers to the research of Xuan and Xuefen (63) and others on the current situation of medical security information network disclosure; the risk perception scale is relatively mature, mainly referring to the influencing factors of risk perception of public health emergencies such as Xie Mengya (64) Research; the measurement of trust perception refers to the research of Mengzhen and Peng (53) and others; the design of the adoption willingness scale mainly refers to the research of scholars on the willingness to use. The specific questionnaire items for each variable are shown in Table 2. Participants who complete the survey will receive a random cash reward to encourage active participation.

**Table 2:** Scale design and sources.

Constructs	Item	Source
Medical insurance information disclosure	I understand the disclosure of medical insurance information	(63)
	I know the methods of medical insurance payment	
	I know what DRG is	
	I know the specific items and scope of medicare reimbursement	
	I understand the fee criteria for each diagnostic-related group	
	I know the medicare process	
	I know the percentage and amount of reimbursement	
Risk perception	I’m concerned about the loss of property due to the lack of transparency of health insurance information	(64)
	I’m concerned about excessive monetary losses due to inaccurate group billing	
	I worry that DRG, a new payment system, is not yet mature	
	I’m worried that DRG-related irregularities will lead to medical disputes	
	I’m concerned that DRG is not objective and reasonable in its costing	
	I’m concerned that DRG does not have enough supervision when paying to reduce the burden	
Trust perception	I think the DRG payment system will save costs	(53)
	I think paying with DRG is more reliable than other payment methods	
	I think the privacy on the DRG payment system is well protected	
	I think it is safe to visit a hospital using the DRG payment system	
	I believe that DRG can standardize medical services and make the medical process more standardized and reliable	
	I think DRG payments ensure that every Medicare fund is spent on the cutting edge	
Willingness to adopt	I accept the DRG payment system	(35)
	I would like to know more about DRG’s payment system	
	I plan to visit a hospital later at a hospital that I paid for using DRG	
	I think there are many benefits to using the DRG payment system	
	I would like to introduce the medical insurance DRG payment system to my friends	

### 3.2. Data collection

Based on the research object and content of this paper, the questionnaire is mainly distributed to patients who have used the medical insurance DRG payment method. From December 20, 2022 to December 30, 2022, the questionnaire is distributed through online channels in the form of online survey. We use the online survey tool Questionnaire to distribute surveys and collect data. Questionnaire Star is the largest professional survey platform in China, with more than 1 million respondents filling out the questionnaire every day, ensuring the integrity and authenticity of the collected information. It specifies a variety of sample attributes such as gender, age, region, occupation, industry, etc., to accurately locate the target group. In addition, various filtering rules, filtering pages, quota control and other conditions can be set, and manual troubleshooting is supported to ensure the validity of its data (65). At the same time, more samples were collected by means of snowball, and 260 valid questionnaires were finally collected (Questionnaire link: https://www.wjx.cn/vm/wo3vFFD.aspx).

Table 3 lists the descriptive analysis of the basic information of the samples. Among the effective samples, male samples accounted for 53.1%, female samples accounted for 46.9%, and the gender composition of the samples was relatively balanced. In terms of age, most of the patients in the sample were 18–30 years old, and the rest of the age groups were evenly distributed. In terms of academic qualifications, undergraduate education accounted for a large proportion, reaching 43.5%. There are many enterprise employees in the sample, accounting for 42.7%. The average monthly income is mostly concentrated in 4,000 ~ 6,000 yuan. In terms of payment methods, only 8.1% of the samples chose to pay at their own expense, indicating that the samples should have a certain degree of understanding of medical insurance payment, which improved the reliability of this survey.

**Table 3:** Basic information descriptive analysis.

Characteristic	Categorization	Frequency	Percent
Gender	Male	138	53.1%
	Female	122	46.9%
Age	18–25 years old	78	30.0%
	26–30 years old	70	26.9%
	31–40 years old	38	14.6%
	41–50 years old	40	15.4%
	51–60 years old	32	12.3%
	Over 60 years	2	0.8%
Education level	High school and below	36	13.8%
	Associate degree	79	30.4%
	Undergraduate	113	43.5%
	Master’s degree and higher	32	12.3%
Career	Government agencies, Institutions	81	31.2%
	Enterprise employee	111	42.7%
	Individual business	39	15.0%
	Farmer	26	10.0%
	Student	3	1.2%
Average monthly income	Below 4,000 yuan	82	31.5%
	4,000–6,000 yuan	108	41.5%
	6,000–8,000 yuan	29	11.2%
	8,000–10,000 yuan	34	13.1%
	More than 10,000 yuan	7	2.7%
Payment method	Medical insurance for urban workers	83	31.9%
	Medical insurance for urban residents	84	32.3%
	New rural cooperative medical care	42	16.2%
	Commercial insurance	30	11.5%
	Own expense	21	8.1%

## 4. Empirical analysis and results

### 4.1. Reliability and validity analysis

In this study, SPSS and AMOS software were used to conduct confirmatory factor analysis to evaluate the reliability and validity of the scale. Previous academic research has found that when the Cronbach’s alpha coefficient of each variable in the model exceeds 0.7 (66), and the combined reliability (CR) is also greater than 0.7, the model meets the reliability test (61). In the confirmatory analysis results in Table 4, the Cronbach’s alpha coefficients of medical insurance information disclosure, risk perception, trust perception and willingness to adopt all exceed 0.7. In addition, the combined reliability (CR) of each variable is greater than 0.7, and the model meets the reliability test.

**Table 4:** Confirmatory analysis results.

Variable	Code	Cronbach’s alpha	CR	AVE	Standard load	S.E.
Medical insurance information disclosure	Q7	0.964	0.964	0.795	0.880	–
	Q8				0.856	0.108
	Q9				0.904	0.106
	Q10				0.874	0.109
	Q11				0.929	0.106
	Q12				0.908	0.104
	Q13				0.888	0.105
Risk perception	Q14	0.977	0.978	0.882	0.995	–
	Q15				0.909	0.038
	Q16				0.940	0.033
	Q17				0.933	0.035
	Q18				0.946	0.032
	Q19				0.908	0.039
Trust perception	Q20	0.907	0.908	0.621	0.835	–
	Q21				0.802	0.091
	Q22				0.803	0.091
	Q23				0.741	0.096
	Q24				0.775	0.091
	Q25				0.769	0.103
Willingness to adopt	Q26	0.902	0.903	0.650	0.815	–
	Q27				0.851	0.092
	Q28				0.784	0.090
	Q29				0.775	0.089
	Q30				0.805	0.093


*χ*^2^/*df* = 2.640; RMSEA = 0.08; NFI = 0.913; IFI = 0.944; CFI = 0.944.

According to previous studies, if the standardized factor loading exceeds 0.70 and the average variance extraction (AVE) is greater than 0.50, the model meets convergent validity (46, 47). In Table 3, the loads of each standardized factor are between 0.741 and 0.995, all greater than 0.70, and the average variance is also between 0.621 and 0.882, all greater than 0.50. The model supports convergent validity.

### 4.2. Correlation analysis and regression analysis

#### 4.2.1. Correlation analysis

This paper mainly analyzes the correlation between medical insurance information disclosure, risk perception, trust perception and adoption willingness, which lays the foundation for further research. The relevant analysis results are shown in Table 5 below.

**Table 5:** Correlation analysis.

	Variable	1	2	3	4	5	6	7	8	9	10
1	Gender	1									
2	Age	0.145	1								
3	Education level	0.165	−0.031	1							
4	Career	−0.094	0.074	0.084	1						
5	Average monthly income	−0.163^*^	−0.068	0.200	−0.002	1					
6	Payment method	−0.034	0.027	−0.009	−0.075	−0.151	1				
7	Medical insurance information disclosure	0.037	−0.006	−0.147	0.167	0.004	−0.043	1			
8	Risk perception	0.003	0.101	0.107	−0.028	0.022	0.024	−0.636^**^	1		
9	Trust perception	−0.004	−0.061	−0.103	0.136	0.053	−0.037	0.749^**^	−0.394^**^	1	
10	Willingness to adopt	−0.052	−0.031	−0.025	0.156	0.029	−0.043	0.626^**^	−0.376^**^	0.782^**^	1

Correlation analysis is to analyze the degree of correlation between various variables studied. In this paper, SPSS 26.0 is used for correlation analysis, and the results are shown in Table 5 (67). Correlation analysis is firstly to verify whether there is correlation between variables, and secondly to examine the degree of correlation between variables. In studies, Pearson correlation coefficient is usually used to describe the degree of correlation. When the absolute value is 0.8–1.0, it can be considered that there is a strong correlation between variables. When the absolute value is 0.6–0.8, it can be considered that there is a strong correlation between variables. When the absolute value is 0.4–0.6, it can be considered that there is a moderate degree of correlation between variables. When the absolute value is 0.2–0.4, it can be considered that there is a weak correlation between variables. When the absolute value is between 0.0 and 0.2, the correlation between variables can be considered to be very weak or no correlation (68).

It can be seen from Table 5 that there is a significant negative correlation between medical insurance information disclosure and risk perception (*r* = −0.636^**^, *p* < 0.05); medical insurance information disclosure and trust perception (*r* = 0.749^**^, *p* < 0.05), it has Significantly positive correlation; Risk perception has a significant negative correlation with adoption intention (*r* = −0.376^**^, *p* < 0.05); Trust perception has a significant positive correlation with adoption intention (*r* = 0.782^**^, *p* < 0.05). Other demographic variables had no or very weak correlation with the study variables.

#### 4.2.2. Regression analysis

It can be seen from Table 6 above that the linear regression analysis takes medical insurance information disclosure as an independent variable and trust perception as a dependent variable, and the regression coefficient value of medical insurance information disclosure is 0.678 (*t* = 18.153, *p* = 0.000 < 0.01), which means The disclosure of medical insurance information will have a significant positive impact on trust perception, assuming that H1 is established.

**Table 6:** Regression analysis of medical insurance information disclosure on trust perception.

	Non standardized coefficient B	Standardization coefficient beta	*t*	*p*	VIF
Constant	1.131	–	7.570	0.000^**^	–
Medical insurance information disclosure	0.678	0.749	18.153	0.000^**^	1.000
*R*^2^		0.561			
Adjusted *R*^2^		0.559			
*F*		*F* = 329.517, *p* = 0.000			
D–W value		2.727			

It can be seen from Table 7 above that the linear regression analysis is performed with medical insurance information disclosure as an independent variable and risk perception as a dependent variable, and the regression coefficient value of medical insurance information disclosure is-0.519 (*t* = −13.236, *p* = 0.000 < 0.01), It means that the disclosure of medical insurance information will have a significant negative impact on risk perception, and the hypothesis H2 is established.

**Table 7:** Regression analysis of medical insurance information disclosure on risk perception.

	Non standardized coefficient B	Standardization coefficient beta	*t*	*p*	VIF
Constant	4.716	–	30.069	0.000^**^	–
Medical insurance information disclosure	−0.519	−0.636	−13.236	0.000^**^	1.000
*R*^2^		0.404			
Adjusted *R*^2^		0.402			
*F*		*F* = 175.204, *p* = 0.000			
D–W value		2.025			

From Table 8 above, it can be seen that using trust perception as an independent variable and adopting willingness as a dependent variable for linear regression analysis, the regression coefficient value of trust perception is 0.779 (*t* = 20.146, *p* = 0.000 < 0.01), which means that trust perception Will have a significant positive impact on the willingness to adopt, assuming that H3 is established.

**Table 8:** Regression analysis of trust perception on adoption willingness.

	Nonstandardized coefficient B	Standardization coefficient Beta	*t*	*p*	VIF
Constant	0.905	–	6.050	0.000^**^	–
Trust perception	0.779	0.782	20.146	0.000^**^	1.000
*R*^2^		0.611			
Adjusted *R*^2^		0.610			
*F*		*F* = 405.878, *p* = 0.000			
D–W value		2.296			

**Figure 2:**
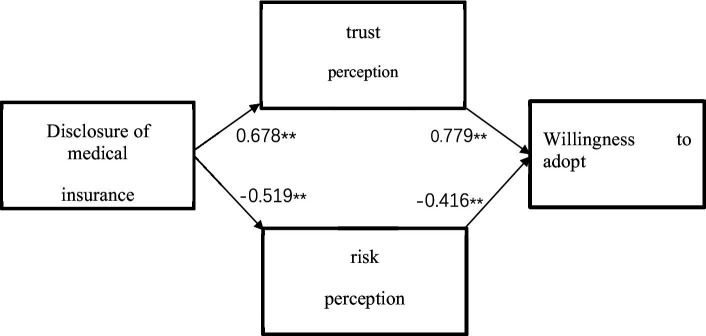
Model result graph.

It can be seen from Table 9 above that the risk perception is used as an independent variable, and the willingness to adopt is used as a dependent variable for linear regression analysis, and the regression coefficient value of risk perception is −0.416 (*t* = −6.519, *p* = 0.000 < 0.01), which means Risk perception will have a significant negative impact on adoption willingness, and hypothesis H4 is established. Finally, the obtained model results are shown in Figure 2.

**Table 9:** Regression analysis of risk perception on adoption willingness.

	Non standardized coefficient B	Standardization coefficient beta	*t*	*p*	VIF
Constant	4.946	–	27.059	0.000^**^	–
Risk perception	−0.416	−0.376	−6.519	0.000^**^	1.000
*R*^2^		0.141			
Adjusted *R*^2^		0.138			
*F*		*F* = 42.491, *p* = 0.000			
D–W value		2.040			

### 4.3. Discussion

In summary, unlike previous scholars who mostly focused on the willingness of medical staff or management to adopt and use the DRG medical payment system, this study conducted an in-depth study from the patient’s perspective, while paying attention to the current situation of inadequate information disclosure about the medical insurance system, and explored the relationship between medical insurance information disclosure, risk perception, trust perception and patients’ willingness to adopt DRG through SPSS empirical analysis based on survey data and relevant theories (69). According to the empirical analysis results, all hypotheses have been effectively verified. Firstly, medical insurance information disclosure has a significant positive impact on patients’ perception of trust and a significant negative impact on patients’ perception of risk. The more comprehensive the information patients have, the stronger their sense of trust will be and the lower their perceived risk will be. In other words, medical insurance information disclosure can improve patients’ perception of trust and reduce their perception of risk. Secondly, patients’ perception of trust has a significant positive impact on their willingness to adopt DRG, while patients’ perception of risk has a significant negative impact on their willingness to adopt DRG. Improving patients’ perception of trust or reducing patients’ perception of risk is conducive to improving patients’ willingness to adopt DRG (70).

## 5. Conclusion

This study found that medical insurance information disclosure can improve patients’ trust, reduce patients’ risk perceptions, and increase patients’ willingness to adopt DRGS. In practical application, the healthcare industry should use various social media to publicize the DRG payment policy and increase the publicity to make patients understand the DRG payment policy (52). In hospital management, hospital managers need to pay attention to the data needs of DRG grouping, organize and count the data sources of DRG grouping, and establish a good DRG-specific database, so as to improve the professionalism and scientificity of statistics, provide comprehensive information for patients, and continuously improve the management level of hospitals (71). At the same time, it is necessary to strengthen the publicity to the public, make the information open, inform the patients of the payment method of medical insurance in time, so that the patients can enjoy the autonomy in the payment method, so that the patients can actively cooperate with the doctors and make reasonable choices in the treatment process, and improve the patients’ willingness to adopt DRGS (54).

### 5.1. Theoretical significance

As a product of the era of medical and health system reform, DRG medical insurance payment reform has affected the development of hospital operation management, budget management, internal control, and performance management to a certain extent. DRG has injected new impetus into the high-quality development of various medical institutions. In order to improve the use effect of the DRG payment system, this study conducts research on the content of medical insurance DRG payment, and makes several theoretical contributions to this field. First, this study focused on patients’ willingness to adopt the DRG payment system. Although there have been more and more studies on medical insurance DRG in recent years, most of them focus on medical institutions and medical insurance funds, and few studies focus on the perspective of patients. However, the essence of the reform of DRG payment methods is to benefit medical insurance Therefore, it is necessary to conduct an in-depth discussion on the perspective of patients, and this study broadens the scope of research in this field (72). Secondly, this study explores the impact of risk perception and trust perception on patients’ adoption of DRG system, which enriches the application of risk perception theory and trust perception theory in the medical field. The research results show that improving patients’ trust perception and reducing patients’ risk perception are conducive to improving patients’ willingness to adopt DRG. In order to improve or reduce these two internal perception states of patients, the key lies in the disclosure of medical insurance information. Finally, this study broadens the application of information disclosure theory in the healthcare field. Information disclosure, as an important tool to reduce information asymmetry, can be effective in reducing users’ risk perceptions. In the process of adopting new health care payments by patient groups, information disclosure should be used to allow patients to clearly understand information related to their own interests, it will achieve the effect of improving patients’ sense of trust and reducing their sense of risk.

### 5.2. Management significance

This study provides some practical implications for various medical institutions in managing medical insurance information disclosure. First of all, all medical institutions should pay attention to the disclosure of medical insurance information and increase their disclosure (33). The disclosure of medical insurance information by medical institutions is an important way for the public to obtain information. It can enable the public to understand medical insurance policies, understand medical insurance benefits more conveniently, and improve patients’ medical experience. Through empirical analysis, this study finds that the disclosure of medical insurance information can enhance patients’ trust perception, reduce their risk perception and thus increase their willingness to adopt DRG medical insurance payment methods. DRG is a new medical insurance payment method. In order to improve patients’ acceptance of DRG, it is recommended that medical institutions increase the disclosure of medical insurance information, especially the disclosure of information related to patients’ interests, and enhance patients’ trust in DRG payment methods. In addition, medical institutions should increase the disclosure rate of medical insurance information. At present, the information disclosure of various medical institutions has the characteristics of selective content. Most of them choose to disclose medical insurance information involving a wide range of people, resulting in incomplete disclosure of information, which is not conducive to the public has a comprehensive understanding of medical insurance policies and services, especially for a new payment system DRG, complete information disclosure is even more important, which can help reduce patients’ risk perception (73). At the same time, this study also highlights the influence of patients’ internal state on the willingness to adopt DRG payment (74). All medical institutions should pay attention to patients’ perception, improve patients’ sense of trust, and reduce their perception of risk (75).

### 5.3. Limitations and prospects

This study has several limitations. First of all, the data in this study are limited to patients in individual medical institutions. Such conclusions may not be accurate enough and have certain limitations. In future research work, the scope of data collection can be effectively expanded to achieve effective testing of model research results. Secondly, this study only explored patients’ willingness to adopt DRG payment from the perspective of medical insurance information disclosure, and patients’ willingness to adopt a new medical insurance payment method is often affected by many aspects, and it can be explored from more dimensions in the future.

## Data availability statement

The raw data supporting the conclusions of this article will be made available by the authors, without undue reservation.

## Ethics statement

Ethical review and approval was not required for the study on human participants in accordance with the local legislation and institutional requirements. Written informed consent from the participants was not required to participate in this study in accordance with the national legislation and the institutional requirements.

## Author contributions

ZS: conceptualization. QZ: methodology. XW: software and validation. All authors contributed to the article and approved the submitted version.

## Conflict of interest

The authors declare that the research was conducted in the absence of any commercial or financial relationships that could be construed as a potential conflict of interest.

## Publisher’s note

All claims expressed in this article are solely those of the authors and do not necessarily represent those of their affiliated organizations, or those of the publisher, the editors and the reviewers. Any product that may be evaluated in this article, or claim that may be made by its manufacturer, is not guaranteed or endorsed by the publisher.
